# A Distinct Saponin Profile Drives an Olfactory-Mediated Aggregation in the Aquacultivated Sea Cucumber *Holothuria scabra*

**DOI:** 10.3390/md21030184

**Published:** 2023-03-16

**Authors:** Emily J. S. Claereboudt, Michel R. Claereboudt, Philippe Savarino, Guillaume Caulier, Loic Gaumez, Magali Deleu, Pascal Gerbaux, Igor Eeckhaut

**Affiliations:** 1 Biology of Marine Organisms and Biomimetics Unit, Research Institute for Biosciences, University of Mons (UMONS), 7000 Mons, Belgiumigor.eeckhaut@umons.ac.be (I.E.); 2 Laboratory of Molecular Biophysics of Interfaces, Gembloux Agro-Bio Tech, University of Liege, 5030 Gembloux, Belgium; 3 Department of Marine Science and Fisheries, College of Agricultural and Marine Sciences, Sultan Qaboos University, Muscat 123, Oman; 4 Organic Synthesis and Mass Spectrometry Laboratory (S2MOs), Research Institute for Biosciences, University of Mons (UMONS), 7000 Mons, Belgium; 5 Belaza Marine Station (IH.SM-UMONS-ULIEGE), Toliara 601, Madagascar; 6 Indian Ocean Trepang, Mahavatsy II, Toliara 601, Madagascar

**Keywords:** saponins, *Holothuria scabra*, aggregation, behavior, pheromone

## Abstract

Intraspecific chemical communication between echinoderms has often been limited to prespawning aggregation. However, sea cucumber farmers have long observed year-round adult aggregation as a potential source of disease propagation and the suboptimal use of available sea pen acreage and food resources. In this study, through spatial distribution statistics, we demonstrated the significant aggregation of the aquacultivated sea cucumber *Holothuria scabra* both as adults in large sea-based pens and as juveniles in laboratory-based aquaria, proving that aggregation in these animals is not only observed during spawning. The role of chemical communication in aggregation was investigated using olfactory experimental assays. Our study established that the sediment that *H. scabra* feeds on as well as the water preconditioned by conspecifics induced positive chemotaxis in juvenile individuals. More specifically, through comparative mass spectrometry, a distinct triterpenoid saponin profile/mixture was identified to be a pheromone allowing sea cucumber intraspecific recognition and aggregation. This “attractive” profile was characterized as containing disaccharide saponins. This “attractive” aggregation-inducing saponin profile was, however, not conserved in starved individuals that were no longer attractive to other conspecifics. In summary, this study sheds new light on the pheromones in echinoderms. It highlights the complexity of the chemical signals detected by sea cucumbers and suggests a role of saponins well beyond that of a simple toxin.

## 1. Introduction

Aggregating behavior is the tendency for organisms (generally of the same species) to group together to form aggregates, which can lead to benefits in species fitness, such as a reduced risk of predation and better access to food resources and/or mates [1]. The aggregation of conspecifics has been widely reported in echinoderms, where it has both been observed in the field and further analyzed in the laboratory. In sea stars, the aggregations of *Asterias rubens* were studied by Moore and Campbell [2], who showed that not only were individual starfish attracted to the waterborne scents of conspecifics but that foraging starfish were more attractive than nonfeeding ones. Aggregation in *A. rubens* may, therefore, be a response to optimize food localization. This is also the case with *Acanthaster planci* [3]. In situ experiments on the brittle star *Ophiothrix fragilis* demonstrated that the animal’s responses to conspecifics were important for maintaining aggregations and, thus, for their optimal localization for suspension feeding [4].

In sea urchins, laboratory experiments demonstrated that the presence of predacious lobster increased the tendency of the urchin *Strongylocentrotus droebachiensis* to aggregate, suggesting that aggregation in this species is an adaptive behavior to predation [5]. When the influence of the spawning group size and the degree of aggregation was investigated with respect to in situ fertilization in the sea urchin *S. franciscanus*, authors found an increase in the group size, and aggregation led to an increase in fertilization success [6]. The aggregation of the tropical Indo-Pacific species *Holothuria scabra* was found to peak a few days before the full moon in situ and often resulted in the formation of spawning pairs, although larger aggregations were also observed [7]. The sea urchins *Paracentrotus lividus* and *S. droebachiensis* form 2D aggregations under stronger water flows and engage more tube feet to enhance tenacity or form tighter intraspecific aggregations to mitigate hydrodynamic forces [8]. Similar behavior has also been observed in the two sea cucumbers *Thyone aurea* and *Pentacta doliolum*, which adopt a commensalistic interaction and group together, enhancing their ability to survive in environments with strong wave action [9].

Overall, four main hypotheses can be put forward to explain aggregation in echinoderms: (i) optimizing feeding [3,4], (ii) defense against predation (safety in numbers) [5], (iii) improving fertilization success at spawning [6,7], and (iv) resisting exposure to strong wave action [8,9]. Although it is widely accepted that most examples of aggregating echinoderms resulted from chemoattraction between conspecifics, there is a striking gap in the current literature that involves a lack of consideration for the precise nature of this chemoattraction.

Chemical cues constitute much of the language of life in aquatic ecosystems [10]. Compounds involved in chemical communication are collectively defined as semiochemicals. This includes both pheromones, which mediate communication between members of the same species, and allelochemicals, which denote chemicals used for communication between individuals from different species [11].

Although echinoderm pheromones have never been identified per se, the induction of spawning [12,13,14,15,16] or the movement of some species in response to the preconditioned water of conspecifics has been reported [16,17]. For example, the sea star *A. rubens* and the sea urchins *Psammechinus miliaris* and *Echinus esculentus* demonstrated statistically significant movement towards the water preconditioned by conspecifics [17]. In holothuroids, olfaction-mediated aggregation has been demonstrated at the time of spawning for *Holothuria arguinensis*. In this Mediterranean species, the water conditioned by males (but not females) attracted both males and females [16]. However, the precise nature of these pheromones remains enigmatic.

Only three recent studies have specifically identified an echinoderm semiochemical. Brasseur et al. [18] characterized sea urchin spinochromes as kairomones (an allelochemical that benefits solely the receiving organism) allowing ectocommensal shrimps to select their host *Echinometra mathaei*. Similarly, in Holothuroidea, triterpenoid glycosides (i.e., saponins) were identified as kairomones involved in the host selection by Harlequin crabs (*Lissocarcinus orbicularis*) of their host sea cucumber *Bohadschia vitiensis* [19] and by pearlfish (*Encheliophis vermicularis*) of their host sea cucumber *Holothuria leucospilota* [20].

Saponins are specialized metabolites commonly known to have a defensive function in the plant kingdom, but they are also abundant in some species of sponges and in all species of holothuroids and asteroids [21].

Saponins are omnipresent in different holothuroid tissues [22] and their surrounding conditioned water [23], with more than 250 chemical species currently described [24]. In addition to the use of saponins by symbiotic crabs [19] and pearlfish [20] as host-identifying kairomones, the cytotoxic and, thus, defensive nature of holothuroid saponins is also well established [25,26].

In the present work, we hypothesize that saponins may also act as pheromones involved in intraspecific chemical communication and aggregation in holothuroids. We first investigated the aggregation of adult and juvenile *H. scabra* in large field-based assays and laboratory experiments. We then considered the precise nature of the chemoattraction taking place using olfactory behavioral assays coupled with mass spectrometry analyses for the chemical characterization of the attractive pheromonal stimuli inducing positive chemotaxis.

## 2. Results

### 2.1. Spatial Distribution of Adults in Sea Pens

**Figure 1:**
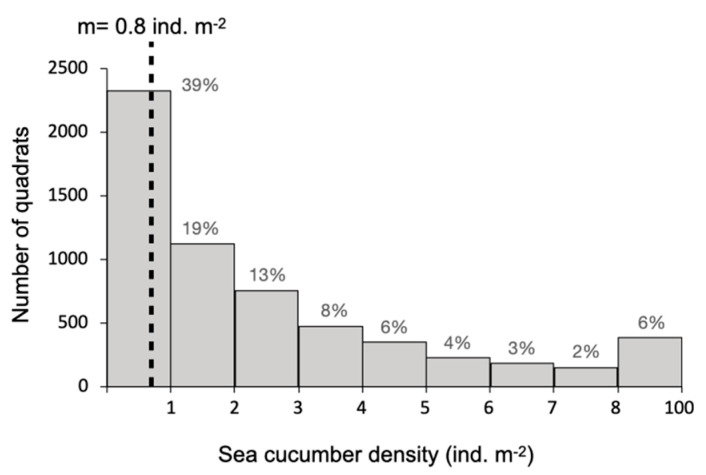
Histogram of sea cucumber density (ind. m^−2^) in considered quadrats. The percentage that each bar represents is indicated above each bar, and the average density is indicated with a dotted line.

A total of 70 surveys were conducted in 10 sea pens over 7 months (January–July 2018). Overall, 6056 *H. scabra* adults were recorded, with 31% found above the sediment and with 69% buried in the sediment. In total, 61% of all the surveyed quadrats were “occupied” by at least one sea cucumber (Figure 1). The maximum number of sea cucumbers observed in a single 4 m^2^ quadrat was 90; however, the average density of sea cucumbers per 4 m^2^ quadrat was 3.37 individuals, i.e., 0.842 ind. m^−2^ (Figure 1).

Out of the 70 surveys conducted, only 9 showed spatial distributions of the sea cucumbers that were not significantly different from a random Poisson process, whereas the 61 remaining surveys indicated a significant overdispersion (i.e, aggregation) of the sea cucumbers in the sea pens (Table 1). This highly significant aggregation was independent of the sea pen considered (two-way ANOVA without replication, *n* = 10, *F*-value = 1.619, *p*-value = 0.133) and of the time of year considered (two-way ANOVA without replication, *n* = 7, *F*-value = 0.7, *p*-value = 0.65). The average number of sea cucumbers above the sediment was significantly lower than the average number of sea cucumbers that were buried (paired *t*-test, *n* = 10, *t* = 5.07, *p*-value = 2.40 × 10^−4^).

### 2.2. Aggregation of Juveniles in Circular Aquaria

**Figure 2:**
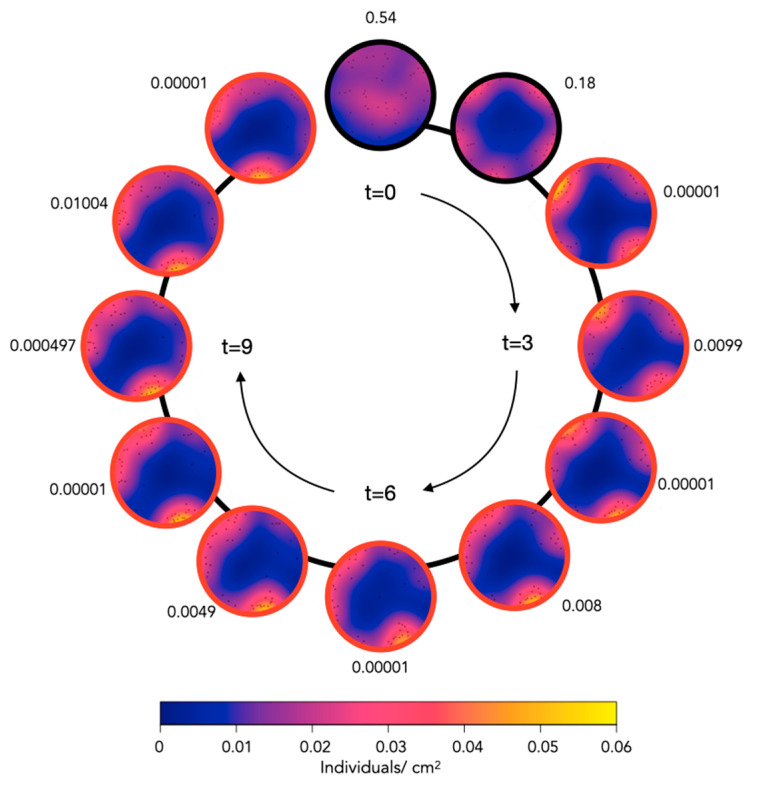
Heat maps of the evolution of sea cucumber density over 12 h. A total of 40 juvenile *H. scabra* sea cucumbers were placed in a circular aquarium (radius of 60 cm) and were photographed hourly over 12 h. *p*-values of the MAD tests are displayed around each experimental circle. Significance (*p* < 0.05) is highlighted with a red border around the experiment. The density heat map is on a scale of 0 to 0.06 individuals per cm^2^.

The movement of 40 *H. scabra* juveniles was tracked over a 12 h time-lapse, and maximum absolute deviation (MAD) tests were calculated for the *X* and *Y* coordinates of the individuals for each time interval (Figure 2). The experimental density was, therefore, 142 juvenile individuals (<100 mm) per meter squared. In addition, to determine the potential influence of the effect of the tank wall, we also considered the angular distribution of the individuals along the circumference of the tank by projecting the position of the animals onto the circumference (data not shown). The MAD test for the angular distribution of the sea cucumber positions projected onto the circumference showed similar results and significant aggregation (data not shown), which allowed us to conclude that the significance of the behavior was independent of the border effect, hence the decision to continue statistical analyses with global spatial aggregation in the tanks.

During the first 2 h following the introduction of the sea cucumbers to the tank, the spatial distribution of the 40 individuals was random, but, following this initial period, the interval was characterized by a significant aggregation every time (*p*-value < 0.05). The heat maps show the initial movement of the individuals toward the wall of the tank, where they distributed randomly (*t* = 1). The individuals then formed three groups, two with more than ten individuals and with a density greater than 0.04 ind cm^−2^. After 6 h, the smaller group with five individuals (and a density of around 0.025 ind cm^−2^) disappeared, its individuals moving towards one of the two bigger groups. For the last three hours of the experiment, most of the individuals remained stationary (Figure 2).

**Figure 3:**
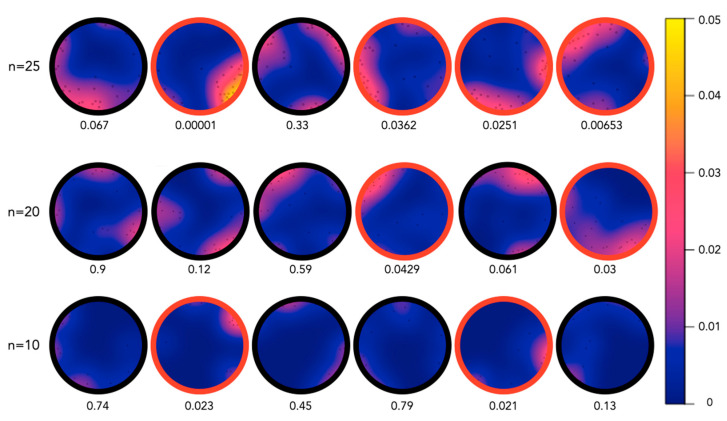
Heat maps of the densities of juvenile sea cucumbers in each overnight experiment. Different numbers of sea cucumbers (*n* = 25, 20, and 10) were photographed in circular aquaria after a 12 h overnight period. The randomness of the spatial distribution of individuals was then analyzed through a maximum absolute deviation test. *p*-values of the tests are displayed under each experiment, and significance (*p* < 0.05) is highlighted with a red border around the experiment. The density heat map is on a scale 0 to 0.05 individuals/cm^2^.

In another experiment, the spatial distribution of 25 juvenile sea cucumbers within a round aquarium (diameter of 60 cm) was observed after 12 h (overnight). The distribution was significantly nonrandom (i.e., the individuals were aggregated) in four out of the six replicates. When 20 sea cucumbers were placed in the experimental tanks, the aggregation was significantly nonrandom in 2 out of 6 replicates, and when a density of 10 sea cucumbers was examined, only 2 out of 6 replicates were a nonrandom spatial distribution of the individuals. In the eight experiments where the distributions were nonrandom (i.e., aggregated), the individuals were often clumped into a single group (Figure 3).

### 2.3. Olfactory Behavioral Assays

**Figure 4:**
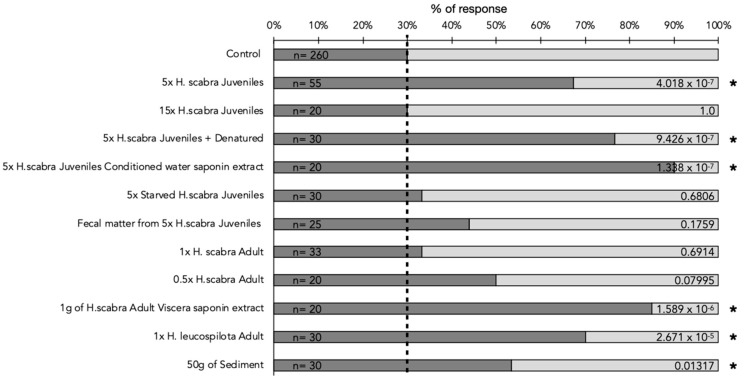
Summary of the response of *H. scabra* juveniles to various types of stimuli tested in a Y-tube olfactometer. Dark grey portions of bars represent the percentage of n *H. scabra* juveniles that moved along the tube during each 10 min experiment. Light grey parts of the bars represent the proportion of individuals that did not move. The number of experiments (*n*) is annotated on the left of the chart for each stimulus. *p*-value of Fisher’s exact test of significance is annotated on the right of the bar graph with an asterisk (*) to indicate significance of movement compared to the control experiments. The proportion of moving and stationary individuals in control experiments in the absence of stimuli is indicated with a vertical dashed line.

To experimentally explore the mechanism behind this observed aggregation among the juveniles, a series of olfactory stimuli were used to induce movement in the sea cucumbers and to narrow down the source and nature of the signal causing the behavior. Overall, over 500 olfactory experiments were conducted (Figure 4). During the control experiments, no olfactory stimuli were present in the experimental setup. The sea cucumbers present in the Y-tube did not display any rheophilic behavior, i.e., they did not move toward the origin of the water current. During the experiments with conspecifics, the motion behavior of the *H. scabra* juveniles varied significantly: The juveniles were attracted significantly by other juveniles when the latter were at a relatively low density (i.e., 5 juveniles of 20 g each placed into 5 L of FFSW—fresh aerated and filtered seawater—for 1 h), and 37 individuals out of 55 (67%) moved towards the stimuli. However, the juveniles were not attracted by an adult conspecific of 350 g placed in 5 L of FFSW for 1 h, and only 11 individuals out of 33 (33%) moved significantly. However, when the density of the juveniles of 20 g reached 15, the movement towards the stimulus became nonsignificant. A statistically significant movement towards the stimuli was also observed when the stimuli were (i) *H. leucospilota* adults, (ii) purified saponin extracts from adult *H. scabra* viscera, (iii) saponin extracted from the water conditioned by *H. scabra* juveniles (3 L of FFSW conditioned for 1 h by five juveniles), (ii) the denatured (i.e., boiled) conditioned water of five *H. scabra* juveniles, or (iv) sediment (food). In all four of these experiments, between 53 and 90% of the individuals were attracted and moved toward the stimuli (i.e., positive chemotaxis) (Figure 4).

The locomotion of the stimulated *H. scabra* juveniles was observed in the glass Y-tube (see figure in supplementary data). An approximate speed of 10 cm/minute was recorded. The locomotion began with the detachment of the terminal quarter of the body’s length, and this was followed by the posterior part of the trivium, which detached from the substrate and contracted and thickened in diameter while the front 3/4 of the body remained thin. This narrowing was certainly due to the contraction of the longitudinal muscle bands at this location and to the relaxation of the circular muscles. A peristaltic wave then progressed from the posterior part to the anterior part, increasing the diameter of the body as the wave progressed. At the level of this peristaltic wave, the podia of the trivium detached themselves from the substrate to reattach themselves to it after the wave. The overall effect of this locomotion was that the animal progressed by about a quarter of its body length after each peristaltic wave.

No significant movement towards the stimuli was observed when the stimuli were (i) starved *H. scabra* juveniles or (ii) fecal matter from *H. scabra* juveniles.

### 2.4. Chemical Analyses of Different Tissues and Extracts

Saponin extractions were conducted and analyzed for healthy/fed and starved *H. scabra* juvenile integument samples in three biological replicates (all of which demonstrated the same composition data). The saponin content of adult *H. scabra* viscera was also analyzed. Previous research on the saponin composition of *H. scabra*-conditioned water conducted by our laboratory was also considered [19].

**Figure 5:**
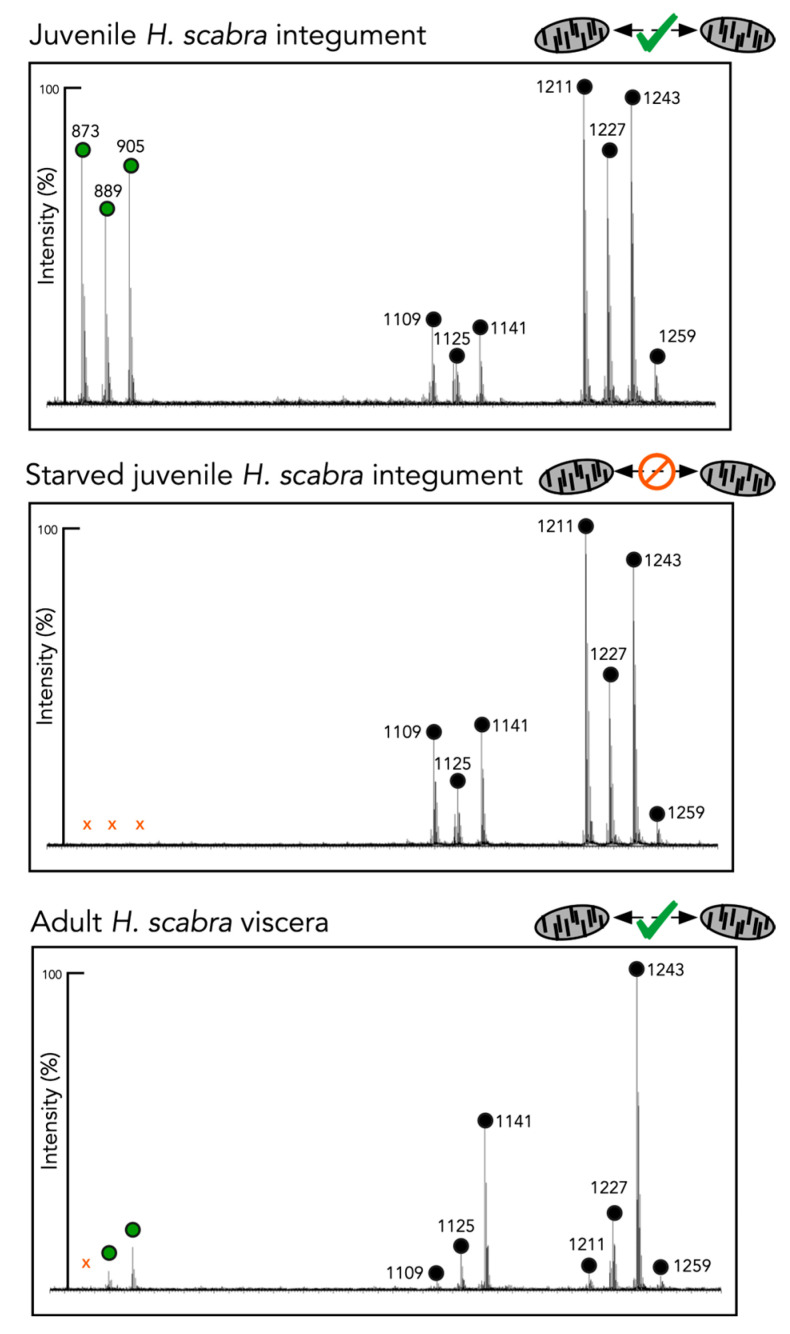
MALDI ToF analysis of different *Holothuria scabra* extracts. Saponin ions (*m*/*z* +Na^+^) are indicated by a circle above their respective peaks. Saponins are named and described in Table 2. Disaccharide saponins are highlighted with a green circle, and stimuli that were attractive during behavioral assays are highlighted with a green tick mark.

Although most (9 out of 10) of the identified saponins (Figure 5) were detected in both the adult and juvenile *H. scabra* tissues, the saponin profiles were very different: Disaccharide saponin ions (*m*/*z* = 873, 889, and 905; Figure 5—green circles) were more abundant in the juvenile tissue than in that of the adults (Figure 5). Differences between the adult viscera tissue extracts and the adult-conditioned water extracts were also observed. The conditioned water lacked *m*/*z* 889 and 905 saponins ions (i.e., with two sugars) (Table 2). Similarly, in the juvenile *H. scabra* tissue, the saponin ions at *m*/*z* 873, 889, and 905 were not observed in the extracts obtained from the animals deprived of food for a week (Figure 5).

The saponin profile from the previous studies conducted in our laboratory [19] and that of *H. leucospilota* [27] were also considered. Adult *H. scabra* showed a lower saponin diversity than the *H. scabra* juveniles, whereas *H. leucospilota* presented the highest saponin diversity, including the two-sugar saponins found only in the healthy juvenile *H. scabra* tissue (Table 2).

In summary, Holothurin B/B4 (*m*/*z* = 905), Holothurin B3 (*m*/*z* = 889), and a third unidentified two-sugar saponin (*m*/*z* = 873) were only detected in the extracts of the stimuli that triggered movement in the *H. scabra* juveniles (Figure 5, Table 2).

## 3. Discussion

Spatial aggregation has long been observed in sea cucumber aquaculture (personal communication from Pr. I. Eeckhaut) but has only been quantified in a handful of wild populations [7,28,29,30,31]. The estimated in situ densities of wild *H. scabra* in a natural ecosystem have been shown to vary from 0.85 to 2.19 ind. 100 m^−2^ [7]. Although a patchy distribution of sea cucumbers has been observed both in wild and aquacultivated populations of sea cucumbers, the phenomena have never been described or quantified in the scientific literature.

In current aquacultural practices, the theoretical density threshold for adult sea cucumbers, set by aquaculturists, is a maximum of 2 adult ind. m^−2^. However, the working density varies a lot in nurseries where ponds or floating *hapas* are used. This limit is therefore 100-fold that of the densities found in wild adult populations. The limit of 2 adults per m^2^ was set considering that (i) the biomass of the *H. scabra* adults obtained per square meter peaks at 700–800 g, which is obtained after 4 to 7 months of rearing [32], and that (ii) the sale of trepang (exported processed dry sea cucumber) is at a minimum of 15 g, which can be obtained from 350 g animals (live weight).

In the present study, we address the spatial distribution of *H. scabra* in the context of aquaculture for the first time. We observed and quantified the significant spatial aggregation of individuals both amongst adults (30 cm long, 400 g) in large aquaculture sea pens and amongst juveniles (<10 cm, 20 g) in small lab-based aquaria.

In the farmed sea pens used in the present study, the density of *H. scabra* was, on average, 0.8 ind. m^−2^ (±1.16 ind. m^−2^) but over a third (42%) of all the quadrats had a density > 2 ind. m^−2^, suggesting that, due to the overall high stocking density of the holothuroids in the sea pens, aggregation behavior resulted in a high proportion of localized densities well above the maximum limit set by aquaculturists.

This recommended theoretical limit of 2 ind. m^−2^, therefore, does not account for the effect of stocking density on aggregation behavior and, in turn, the effect of such aggregation on biomass production. This seemingly simple behavior can lead to the propagation of diseases [33,34] and to the patchy use of acreage, thus leading to greater losses and suboptimal growth rates. In the context of aquaculture, further research on how these animals have optimized their foraging behavior to high aquaculture densities and on if and how this aggregation behavior affects the growth rates, survival rates, and global yield of sea pens is needed.

During the laboratory-based aggregation experiments on *H. scabra* juveniles, we studied the behavior of the juveniles at densities of 10–40 individuals in an aquarium of 0.3 m^2^ (i.e., 35–140 ind. m^−2^). The experimental conditions were also about 100 times higher than the field-based surveys, which observed the densities of wild juveniles (<100 mm) to be around 35 ind. 100 m^2^ [7], while current aquaculture practices recommend a density of 20 ind. m^−2^.

These experiments highlighted highly significant aggregation behavior in the juveniles, particularly at higher stocking densities (70 and 140 ind. m^−2^). Through observing the movement of the individuals in a time-lapse sequence, we could initially observe the individuals moving toward the borders of the tank followed by the formation of distinct clusters. Once the clusters were formed, little change was observed in the spatial distribution for the remaining time of the experiment.

Overall, based on both series of experiments on the spatial distribution of adults and juveniles, a significant aggregation behavior was quantified. These findings will be indispensable for further studies on foraging behavior and for the optimization of aquacultural practices.

Although the aggregation of conspecifics is widely reported in echinoderms and although it is widely accepted that most examples of aggregating echinoderms result from a chemoattraction between conspecifics, i.e., the use of pheromones [12,13,14,15,16], the precise nature of this chemically mediated behavior remains unclear.

**Figure 6:**
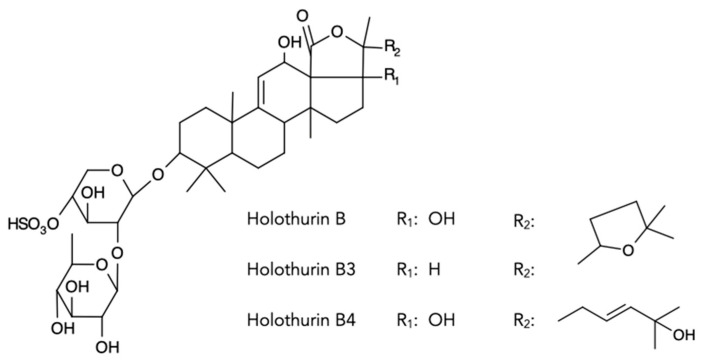
Saponin structures of the disaccharide saponins that were unique to extracts that were attractive to *H. scabra* juveniles during olfactory behavioral assays.

Sea cucumbers are known to secrete species-specific cocktails of saponins [24,35], which can even be shown to be so distinct that saponin profiles can be used as an accurate chemotaxonomic fingerprint, especially in the *Holothuria* genus [35]. In this study, we found that both the saponin-enriched extracts from the conditioned water of the juveniles and the boiled conditioned water significantly attracted conspecifics, eliminating the possible role of thermosensitive proteins that may have contaminated the saponin-enriched extracts. These findings are consistent with saponins being the chemical signal used during conspecific communication in *H. scabra* juveniles, as they are known to be thermostable [36]. The comparison of the mass spectra of the saponin extracts that were determined to be attractive compared to those that were not, clarified the unexpected attraction of the *H. scabra* juveniles towards the *H. leucospilota* adults but not towards the *H. scabra* adults. The saponin profile of *H. leucospilota* was significantly more diverse and contained several saponins in common with the *H. scabra* juveniles, but that was absent in the *H. scabra* adults. The disaccharide saponins Holothurin B3 (*m*/*z* 889) and Holothurin B/B4 (*m*/*z* 905) were unique to all the profiles that were attractive to the *H. scabra* juveniles (Figure 6). These unique saponins were not detected in the *H. scabra* juveniles that had been starved for a week, suggesting a direct link between saponin biosynthesis and diet and/or stress.

Although we cannot claim these precise disaccharide saponins to indeed be pheromones without purifying them and testing their individual properties on *H. scabra*, our results suggest that a very distinct profile of saponins acts as pheromones, either as a distinct mixture or through a unique subset of this mixture of saponins. In addition, the positive attraction of the juveniles towards the conspecific water that was conditioned by 5 juveniles and the loss of this attraction when the same volume of water was conditioned by 15 individuals suggests that the attractiveness of the pheromone is concentration-dependent and that it becomes repellent at high concentrations.

The juveniles were also attracted to the odor of their food. This suggests that the selective feeding behavior of *H. scabra*, which was previously observed [37,38], is olfactory-mediated. *H. scabra* selectively prefers sediments rich in organic matter, and the bioturbation of their feeding activity releases the nutrients trapped in the sediments to benthic ecosystems, a keystone phenomenon in coral reef ecosystem health [39].

Decisions related to foraging for food are among the most critical for an animal’s survival [10]. Animals face a critical decision on how to use either pheromones and/or other odors to orientate their search for new food sources [40]. Deciding when and where to go to find these new food sources requires the animal to integrate information about food availability with cues signaling the presence of food or the presence of other individuals (e.g., pheromones). This, however, implies the determination of whether pheromones point towards a resource supporting growth and reproduction or an already exploited one [41]. In the model nematode *Caenorhabditis elegans*, an inversion of the effect of such pheromones was observed. Bello et al., [41] found that the time at which a worm leaves a food patch affects the animal’s preference for its conspecific pheromones. Roundworms that left early were attracted to their conspecific pheromones, while worms leaving later were repelled by these same pheromones. This seems to be a strategy to optimize food intake during foraging and illustrates how the same pheromone signal can be both attractive and repellent depending on the situation.

Our findings suggest that the foraging behavior of sea cucumbers may also be more complex than previously believed. We hypothesize that a dual foraging behavior may be occurring within *H. scabra* juveniles and perhaps also in adults. In our study, we concluded that sediment, the food of the detritivorous *H. scabra*, was attractive as well as conspecific pheromones but only at relatively low concentrations. The observed concentration-dependent activity of the distinct pheromone is ecologically coherent, as low concentrations could indicate reliable resources (at least if the source sea cucumber has fed); however high concentrations could imply overcrowding and competition. This type of dual foraging has also been described in the corallivore sea star *A. planci*, where feeding individuals will often move towards coral colonies already being fed on rather than patches of coral yet to be touched, suggesting an attraction of conspecifics [3]. In another study on the aggregative pheromone phenyl acetonitrile (PAN) secreted by mature male desert locusts (*Schistocerca gregaria*), the authors showed that *S. gregaria* males demonstrated a concentration-dependent response. Locusts preferred to be within the PAN-permeated air column at low relative doses of the pheromone but away from PAN at high relative doses of PAN [42].

Overall, the aggregation of *H. scabra* juveniles is clearly an olfactive behavior associated with a distinct saponin profile. This along with the olfactory detection of organic matter from food surely play a role in a complex foraging behavior, which warrants further investigation.

To conclude, the present study has confirmed and quantified an aggregation behavior in the aquacultivated sea cucumber *H. scabra* both in adults in large sea pens and in juveniles in controlled circular aquaria. The attraction of the juveniles to conspecific juveniles during olfaction assays allowed us to conclude that this aggregation is an olfactory-mediated behavior. We showed that the *H. scabra* juveniles not only responded to food but also to the odor of conspecific juvenile sea cucumbers as well as saponin-enriched extracts in a concentration-dependent manner. By comparing the saponin compositions of the tissues from the stimuli that were attractive (*H. scabra* juveniles, *H. leucospilota*, and saponin extracts) with those that were not (*H. scabra* adult-conditioned water and starved juveniles), we were able to highlight a distinct saponin profile, including two disaccharide saponins that were only present in the extracts of the “attractive” stimuli.

Saponins are structurally diverse, and profile mixtures are species- and age (juvenile vs. adult)-specific. The present study suggests a role of saponins well beyond that of a simple toxin. In addition to being defensive allomones and intraspecific kairomones, our findings prove that saponins also act as pheromones, further suggesting that the evolutionary expansion of saponin diversity in sea cucumbers was perhaps not solely due to their cytotoxic/defensive nature but also due to their solubility and potential as species-specific semiochemicals.

## 4. Materials and Methods

### 4.1. Organisms

A total of approximately 1000 (<10 cm, 25 g) *Holothuria (Metriatyla) scabra* Jaeger juveniles were used, and 1833 were kindly provided by Indian Ocean Trepang (IOT), Tulear, Madagascar. Most individuals were not sacrificed during this study and were returned to the IOT farm after assays. The IOT farm raises sea cucumber embryos produced by their hatchery to marketable size and weight (more than 350 g) in 12 to 18 months. Their method for growing *H. scabra* includes three successive phases that each require specialized infrastructures (internal aquaria, external tanks, and sea fences) related to the animal size (or weight). Internal aquaria are set up in a hatchery. They each contain 3000 L of seawater, where larvae and small juveniles grow up to a length of 1 cm. Sea cucumbers are then transferred to external tanks (nursery), where they are maintained until they reach a minimum length of 6 cm (c.a. 15 g). This minimum size is adequate for allowing individuals to survive in sea pens (grow-out sites). The nursery of IOT is equipped with around 50 ponds of 100 m^2^ (for juveniles coming out of the hatchery) and 50 other ponds of 1000 m^2^ (for the largest juveniles). Approximately 1000 of the largest juveniles were used in olfactory experiments. Upon arrival at the laboratory at the Institute of Halieutic and Marine Sciences (IH.SM; Toliara, Madagascar), juveniles were placed into 1 m^3^ basins filled with fresh filtered (1 μm) and oxygenated seawater (26 °C and 35‰ salinity) with a 3 cm layer of their dietary sand (same source as the sand used for rearing at IOT) for 2 days to allow for acclimatization. Approximately 10 adult specimens of *H. scabra* (>25 cm, 350 g) were also provided by IOT. Adults came from sea pens in the bay of Sarodrano, which is a grow-out site named the “Mangrove”. At the time of the experiments (January–July 2018), the site included 60 hectares of 17 enclosures, which were 2 to 12 ha each, and 10 enclosures were considered for surveying. Another species, *Holothuria (Mertensiothuria) leucospilota* (*n* = 10), was used in the olfactory experiments (see here below). The adult individuals (>25 cm, 300 g) were hand-collected from the shallow sandy bottom of the Great Reef of Toliara at low tide (depth < 2 m).

### 4.2. Spatial Distribution of Individuals in Sea Pens

**Figure 7:**
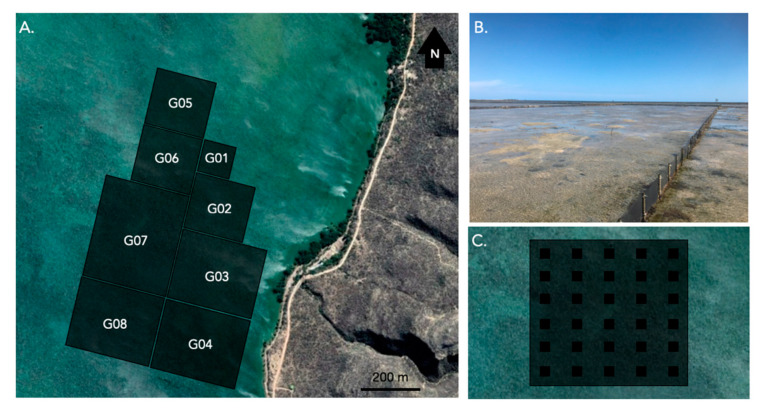
Sea pens used for large-scale spatial distribution analyses of *H. scabra* adults. (**A**) Map of the position of the sea pens analyzed and located in the bay of Sarodrano near Toliara in Southwest Madagascar. (**B**) An example of a sea pen at low tide. (**C**) Example of the regular distribution of the 4 m^2^ quadrats (black squares) used to count the surface and buried adult *H. scabra* sea cucumbers in a sea pen (represented as grey rectangles G01–G08).

Every month for 7 months, between January and July 2018, a team of 4 technicians from IOT carried out an analysis of the distribution and density of sea cucumbers in the sea pens by foot using the quadrat method. At each full moon, at low tide, at depths of less than 1.5 m, in each of the 10 considered sea pens (G01–G08, G10, G11), 30 quadrats of 4 m^2^ were arranged in a systematic grid (Figure 7). For each quadrat, the sea cucumbers visibly present on the substrate were counted first. *H. scabra* is, however, a burrowing species, burying itself until invisible under sandy substrates [43,44]. The substrate in each quadrat was therefore excavated to a depth of 40 cm to identify the buried individuals. A total of 70 distribution maps of sea pens were obtained (10 enclosures × 7 months). For each distribution map, the mean (*Mean*) and variance (*Var.*) of the number of both surface and buried sea cucumbers per quadrat was calculated. Under the null hypothesis of a random Poisson process the statistic “*t*” approximately followed a *t*-distribution with *n* − 1 degrees of freedom, with *t* given by the following equation:

$$t=\frac{\left(\frac{Var.}{Mean}-1\right)}{\sqrt{\frac{2}{n-1}}}$$

Values of *t* higher than *t*_(0.975,2)_ suggested overdispersion of individuals and not a random distribution. This is equivalent to a patchy distribution and the formation of aggregates.

### 4.3. Aquaria Aggregation Experiments

**Figure 8:**
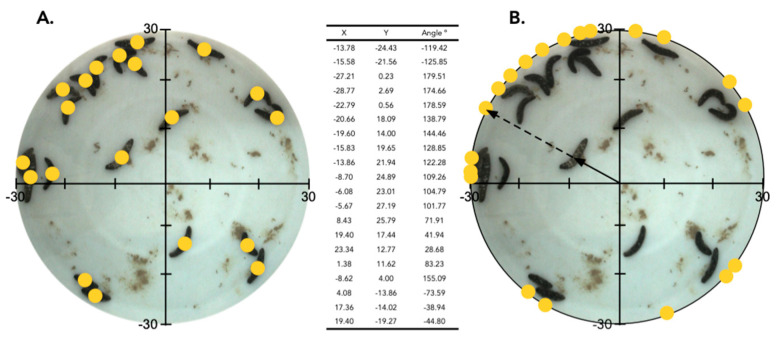
Raw data collection from circular tank aggregation experiments. (**A**) Example of raw data collection from photographs of a circular aquarium containing 20 *H. scabra* juveniles. Using the software Fiji [44], the diameter of the tank was set to 60 cm, and the *X* and *Y* coordinates of the position of each cucumber were measured. (**B**) Example of how sea cucumber positions were projected onto the circumference of the tanks. The coordinates and angles shown in the two photos were the basis of further statistical tests.

In December 2020, circular aquaria of a diameter of 60 cm were prepared with fresh aerated and filtered seawater (FFSW) (28 °C and 35‰ salinity). *H. scabra* juveniles were randomly placed into the aquaria in the evening and were photographed the following morning (12 h later). The *X* and *Y* coordinates of each sea cucumber were determined using the software Fiji [45] (Figure 8). Different densities of sea cucumbers were used, i.e., 25 individuals, 20 individuals, and 10 individuals, and experiments were repeated 6 times with different individuals (a total of 330 individuals).

These sea cucumber densities were based on current aquaculture practices in sea cucumber “nurseries”, where juveniles are reared. There is limited literature on the wild population densities of *H. scabra* juveniles. In the Solomon Islands, Mercier et al. [7] observed densities of wild juveniles (<100 mm) to be around 35 ind. 100 m^2^. In aquaculture nurseries, starting densities vary greatly from a few individuals (e.g., [32,46,47,48]) to more than 1000 per square meter [49,50], and the density varies greatly depending on the size of the juveniles and the system used.

In addition, a different dataset was generated from a time-lapse series of photographs taken over 12 h (7 a.m.–7 p.m.). For this, 40 *H. scabra* juveniles were placed randomly in a circular aquarium with a diameter of 60 cm and were photographed every hour. The spatial distribution of the juvenile sea cucumbers was analyzed using a maximum absolute deviation test [51]. The test is based on comparing the observed number of events within an increasing distance from any events (Ripley’s K function) to a series of Monte Carlo simulations under the null hypothesis of random spatial distribution. This MAD test was applied to the *X* and *Y* coordinates of the spatial distribution as well as to the angular position/distribution of sea cucumbers on the circumference of the tank [52,53] (Figure 8B). The MAD test measures the maximum deviation between the observed and the simulated K-functions and was shown to be a valid inferential test for spatial randomness [54]. The analysis was carried out with R [55] and the spatstat package [51].

### 4.4. Olfactory Behavioral Assay Experimental Setup

**Figure 9:**
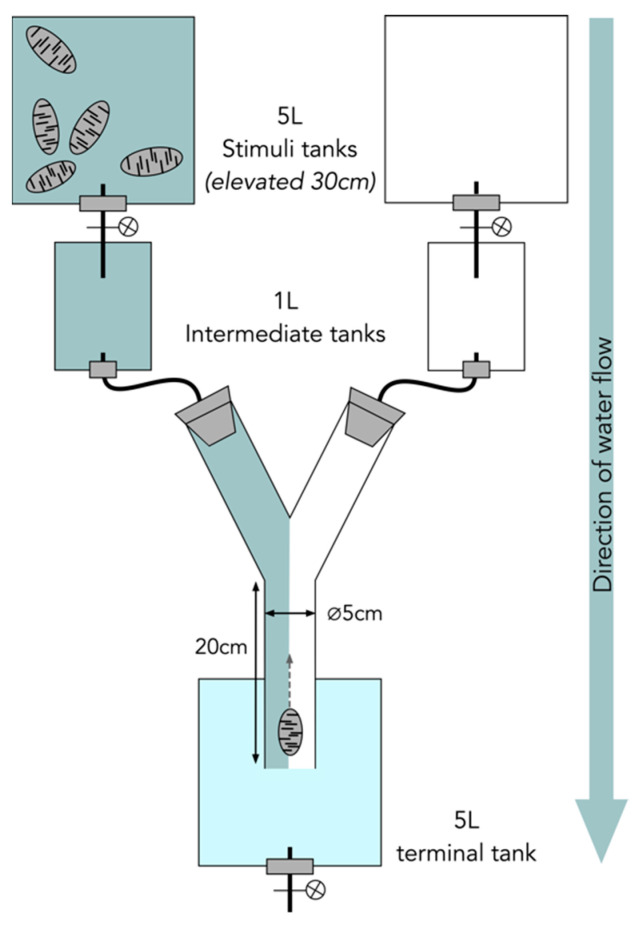
Scheme of the experimental setup of olfactory behavioral assays. Thanks to the elevated stimulus tanks and the adjustment of the three taps (one on each stimulus tank, and one on the terminal tank), a laminar flow through the Y-tube was obtained. The stimulus flow in the olfactometer is represented by grey color.

Assays were conducted during three scientific missions to Madagascar in November 2018, April 2019, and November 2019. A behavioral olfactometer was constructed according to previous studies [19,56,57,58,59] with a glass “Y” shaped tube with a diameter of 5 cm (Figure 9). The tube was attached to two “intermediate tanks” at each end of the Y-tube arms. These intermediate tanks were fed by a controlled flow rate (thanks to taps) of water from stimulus tanks. These 5 L stimulus tanks contained the different stimuli (listed below) or simply water in the case of control experiments (Figure 9). During experiments, laminar flow within the tube was monitored using the dye fluorescein and was adjusted to 2–3 cm/s using the three taps of the experimental setup (Figure 9). The experimental setup was thoroughly cleaned between experiments with fresh water and a cloth to remove any potential residues (e.g., pheromonal track) left by the previously tested sea cucumbers. At least 20 repetition experiments were conducted with 20 different juvenile individuals for each stimulus, and repetitions were limited to 20 for experiments using purified extracts due to limited quantities. Each experiment took 10 min. Individuals were placed headfirst at the beginning of the Y-tube and were not used inside the tube more than once, and the position of the stimuli (left or right) was switched regularly. Control experiments were conducted regularly with only pure seawater, where no stimulus was present. The movement of each of the almost 600 individual juvenile sea cucumbers along the tube was recorded. It should be noted that individuals very rarely moved all the way up into one of the two paired arms (<15% of experiments) and usually stopped at the intersection of the Y-tube. The results analyzed and discussed below, therefore, focus on the absence or presence of movement in the response to stimuli and not the directionality.

Fisher’s test was carried out with R [55] to determine if movement in “stimuli” behavioral assays was significantly different (i.e., independent) from the movement observed in “control” experiments.

The following stimulus solutions were prepared and then used in the experimental setup described above:

- *H. scabra* juvenile-conditioned water: a total of 5 juveniles of 15 g each were placed into 5 L of FFSW for 1 h.
- High-concentration *H. scabra* juvenile-conditioned water: a total of 15 juveniles were placed into 5 L of FFSW for 1 h.
- *H. scabra* adult-conditioned water: one adult of *H. scabra* (350 g) was placed into 5 L of FFSW for 1 h.
- Diluted *H. scabra* adult-conditioned water: One adult of *H. scabra* (350 g) was placed into 5 L of FFSW for 1 hour. 2.5 L of this conditioned water was then diluted twice.
- *H. leucospilota* adult-conditioned water: one adult of *H. leucospilota* (250 g) was placed into 5 L of FFSW for 1 h.
- Conditioned water of starved *H. scabra* juveniles: A total of 5 juveniles were placed into 5 L of FFSW without food for 1 week prior to the experiment, and oxygenation was maintained. Prior to experiments, 5 L of water was conditioned by the 5 starved individuals for 1 h.
- Denatured conditioned water of *H. scabra* juveniles: a total of 5 L of FFSW was conditioned for 1 hour with 5 juveniles, and the water was then boiled for 30 min before being used in experiments.
- Conditioned water of fecal matter of *H. scabra* juveniles: A total of 5 juveniles were placed into 5 L of FFSW overnight without food. The next day, the animals were removed, and the water changed whilst maintaining the fecal matter in the aquarium. The FFSW water was conditioned with the fecal matter for one hour before being used in experiments.
- Saponin extract of adult *H. scabra* viscera: Saponin extraction and purification were performed using the previously described method [60] on *H. scabra* viscera at UMons, Belgium. In brief, an overnight maceration of dry ground intestinal tissue in 70% methanol was conducted. The extract was then filtered and partitioned sequentially against hexane, dichloromethane, and chloroform. The methanol extract was then dried, resuspended in isobutanol, and partitioned against water to remove salts. The isobutanol fraction was then dried and resuspended in milli-Q water for further analyses or experiments. During the olfactory experiments, an approximate concentration of 200 mg L^−1^ was used, corresponding approximately to the quantity of saponin in sea-cucumber-conditioned water [61].
- Saponin extract of *H. scabra* juvenile-conditioned water: A total of 3 L of FFSW conditioned for 1 H by 5 juveniles was passed through a column of Amberlite XAD-4 (Sigma–Aldrich, St. Louis, MO, USA) as previously described [19,60,61]. Although this method is limited in its extraction ability, this is the selected method for in-field extractions, as solvents are limited. A concentration of 200 mg L^−1^ was used.
- Sediment-/food-conditioned water: A total of 50 g of the sediment used to feed the sea cucumbers in inland growing basins of IOT was placed into 5 L of FFSW. The water was conditioned with the sediment for 1 H before being used in olfactory experiments.

### 4.5. Mass Spectrometry Analyses

Saponins were extracted using the liquid partitioning method described above [60] from 5 g of viscera of *H. scabra* adults, from the entire integument of *H. scabra* juveniles, and from the entire integument of starved *H. scabra* juveniles. The dried extracts were analyzed through mass spectrometry to identify the saponin profiles. Analyses were performed using a Waters Q-ToF Premier mass spectrometer equipped with a matrix-assisted laser desorption/ionization ion source (MALDI) in positive mode. All the detected ions correspond to [M + Na]^+^ adducts. The MALDI source consisted of a Nd:YAG solid-state laser (355 nm) with a maximum pulse energy of 104.1 µJ delivered in 2.2 ns to the analysis spot at a frequency of 125 Hz. The matrix consisted of a mixture of di-hydrobenzoic acid (25 mg) and dimethylaniline (6 µL, 99.9%) in 250 µL of acetonitrile/water (*v*/*v*). A droplet of matrix solution (1 µL) was spotted on a stainless steel plate and was air-dried before depositing a droplet (1 µL) of the sample solution (1 mg of dried extract in 1 mL of water). For recording of the single-stage MALDI-MS(+) spectra, all the ions were analyzed using the ToF analyzer, operating at a 1 s integration time [62].

## Figures and Tables

**Table 1:** Summary of the degree of aggregation observed in the 70 surveys carried out in the 10 sea pens. Using thirty 4 m^2^ quadrats per sea pen, surface and buried animals were counted as described in the methodology. Surveys were considered to have significantly aggregated spatial distributions if the *p*-value of the *t*-test of Variance/Mean was less than 0.05.

	Number of Significantly Aggregated Spatial Distributions among 70 Surveys
Buried animals	55 (78%)
Surface animals	46 (66%)
Both buried and surface animals	61 (87%)

**Table 2:** Summary of saponin content of different stimuli tested in olfactory assays. In the present study, saponin content was determined for both healthy and starved juvenile *H. scabra* integument tissues as well as for adult *H. scabra* viscera. These compositions were compared to previous findings on the conditioned water of *H. scabra* adults [19] and the body-wall mucus (BW) of *H. leucospilota* [27]. In association with Figure 5, columns in dark gray are associated with stimuli that did not induce significant movement in *H. scabra* juveniles. Columns in light gray represent those that induced significant movement. Some *m*/*z* are associated with several potential isomeric structures.

	[M + Na]^+^ *m*/*z*	No. of Sugar Residues	*H. scabra* Juvenile-Integument	*H. scabra* Juvenile Starved Integument	*H. scabra* Adult Viscera	*H. scabra* Adult Conditioned Water	*H. leucospilota* BW Mucus
Holothurinoside N	1317.1	5					X
Holothurinoside M	1301.6	5					X
Girseaside A	1289.6	5					X
Impatinside B	1271.7	5					X
Holothurin A3	1259.3	4	X	X	X		X
Scabraside B Holothurin A 17- or 25- dehydroechinoside A	1243.3	4	X	X	X	X	X
Holothurin A2	1229.5	4				X	
Scabraisde A Fuscocineroside B/C 24-dehydroechinoside A	1227.5	4	X	X	X	X	X
Unidentified	1211	4	X				
Desholothurin A Desholothurin A1	1141.5	4	X	X	X		X
Holothurinoside C	1125.6	4	X	X		X	
Unidentified	1109	4	X	X			
Leucospilotaside A	921.4	2					X
Holothurin B4 Nobiliside B Holothurin B	905.4	2	X		X		X
Holothurin B3	889.4	2	X		X		X
Unidentified	873	2	X				

## Data Availability

The data presented in this study are available on request from the corresponding author.

## Supplementary Materials

The following supporting information can be downloaded at: https://www.mdpi.com/article/10.3390/md21030184/s1, Figure S1: Locomotion of *H. scabra* juvenile.

## References

[1] Philippe A.-S., Jeanson R., Pasquaretta C., Rebaudo F., Sueur C., Mery F. (2016). Genetic Variation in Aggregation Behaviour and Interacting Phenotypes in Drosophila. Proc. R. Soc. Lond. Ser. B Biol. Sci..

[2] Moore R.J., Campbell A.C., Keegan B.F., O’Connor B.D.S. (1984). An Investigation into the Behavioural and Ecological Bases for Periodic Infestations of *Asterias rubens*. Echinodermata.

[3] Ormond R.F.G., Campbell A.C., Head S.H., Moore R.J., Rainbow P.R., Saunders A.P. (1973). Formation and Breakdown of Aggregations of the Crown-of-Thorns Starfish *Acanthaster Planci* (L.). Nature.

[4] Broom D.M. (1975). Aggregation Behaviour of the Brittle-Star *Ophiothrix fragilis*. J. Mar. Biol. Assoc. UK.

[5] Bernstein B.B., Schroeter S.C., Mann K.H. (1983). Sea Urchin (*Strongylocentrotus Droebachiensis*) Aggregation Behavior Investigated by a Subtital Multifactorial Experiment. Can. J. Fish. Aquat. Sci..

[6] Levitan D.R., Sewell M.A., Chia F.-S. (1992). How Distribution and Abundance Influence Fertilization Success in the Sea Urchin *Strongylocentrotus franciscanus*. Ecology.

[7] Mercier A., Battaglene S.C., Hamel J.-F. (2000). Periodic Movement, Recruitment and Size-Related Distribution of the Sea Cucumber *Holothuria Scabra* in Solomon Islands. Hydrobiologia.

[8] Frey D.L., Gagnon P. (2016). Spatial Dynamics of the Green Sea Urchin *Strongylocentrotus droebachiensis* in Food-depleted Habitats. Mar. Ecol. Prog. Ser..

[9] Barkai A. (1991). The Effect of Water Movement on the Distribution and Interaction of Three Holothurian Species on the South African West Coast. J. Exp. Mar. Biol. Ecol..

[10] Brönmark C., Hansson L.-A. (2012). Aquatic Chemical Ecology. Chemical Ecology in Aquatic Systems.

[11] Kost C., Jørgensen S.E., Fath B.D. (2008). Chemical Communication. Encyclopedia of Ecology.

[12] Mercier A., Hamel J.-F. (2002). Perivisceral Coelomic Fluid as a Mediator of Spawning Induction in Tropical Holothurians. Invertebr. Reprod. Dev..

[13] Soong K., Chang D., Chao S.M. (2005). Presence of Spawn-Inducing Pheromones in Two Brittle Stars (Echinodermata: Ophiuroidea). Mar. Ecol. Prog. Ser..

[14] Zacarías-Soto M., Olvera-Novoa M.A., Pensamiento-Villarauz S., Sánchez-Tapia I. (2013). Spawning and Larval Development of the Four-Sided Sea Cucumber, *Isostichopus Badionotus* (Selenka 1867), under Controlled Conditions. J. World Aquac. Soc..

[15] Caballes C.F., Pratchett M.S. (2017). Environmental and Biological Cues for Spawning in the Crown-of-Thorns Starfish. PLoS ONE.

[16] Marquet N., Hubbard P.C., da Silva J.P., Afonso J., Canário A.V.M. (2018). Chemicals Released by Male Sea Cucumber Mediate Aggregation and Spawning Behaviours. Sci. Rep..

[17] Campbell A.C., Coppard S., D’Abreo C., Tudor-Thomas R. (2001). Escape and Aggregation Responses of Three Echinoderms to Conspecific Stimuli. Biol. Bull..

[18] Brasseur L., Caulier G., Lepoint G., Gerbaux P., Eeckhaut I. (2018). Echinometra Mathaei and Its Ectocommensal Shrimps: The Role of Sea Urchin Spinochrome Pigments in the Symbiotic Association. Sci. Rep..

[19] Caulier G., Flammang P., Gerbaux P., Eeckhaut I. (2013). When a Repellent Becomes an Attractant: Harmful Saponins Are Kairomones Attracting the Symbiotic Harlequin Crab. Sci. Rep..

[20] Miyazaki S., Ichiba T., Reimer J.D., Tanaka J. (2014). Chemoattraction of the Pearlfish *Encheliophis vermicularis* to the Sea Cucumber *Holothuria leucospilota*. Chemoecology.

[21] Claereboudt E.J.S., Caulier G., Decroo C., Colson E., Gerbaux P., Claereboudt M.R., Schaller H., Flammang P., Deleu M., Eeckhaut I. (2019). Triterpenoids in Echinoderms: Fundamental Differences in Diversity and Biosynthetic Pathways. Mar. Drugs.

[22] Demeyer M., Wisztorski M., Decroo C., Winter J.D., Caulier G., Hennebert E., Eeckhaut I., Fournier I., Flammang P., Gerbaux P. (2015). Inter- and Intra-Organ Spatial Distributions of Sea Star Saponins by MALDI Imaging. Anal. Bioanal. Chem..

[23] Van Dyck S.V., Caulier G., Todesco M., Gerbaux P., Fournier I., Wisztorski M., Flammang P. (2011). The Triterpene Glycosides of *Holothuria forskali*: Usefulness and Efficiency as a Chemical Defense Mechanism against Predatory Fish. J. Exp. Biol..

[24] Bahrami Y., Franco C. (2016). Acetylated Triterpene Glycosides and Their Biological Activity from Holothuroidea Reported in the Past Six Decades. Mar. Drugs.

[25] Kamyab E., Rohde S., Kellermann M.Y., Schupp P.J. (2020). Chemical Defense Mechanisms and Ecological Implications of Indo-Pacific Holothurians. Molecules.

[26] Eeckhaut I., Caulier G., Brasseur L., Flammang P., Gerbaux P., Parmentier E. (2015). Effects of Holothuroid Ichtyotoxic Saponins on the Gills of Free-Living Fishes and Symbiotic Pearlfishes. Biol. Bull..

[27] Sroyraya M., Kaewphalug W., Anantachoke N., Poomtong T., Sobhon P., Srimongkol A., Suphamungmee W. (2018). Saponins Enriched in the Epidermal Layer of *Holothuria leucospilota* body Wall. Microsc. Res. Tech..

[28] Chantal C. (1989). Les Holothuries Aspidochirotes Du Lagon de Nouvelle Calédonie: Biologie, Écologie et Exploitation.

[29] James D.B. (1994). Seed Production in Sea Cucumbers. Aqua Int..

[30] Lawrence A.J., Ahmed M., Hanafy M., Gabr H., Ibrahim A., Gab-Alla A. (2016). Status of the Sea Cucumber Fishery in the Red Sea, the Egyptian Experience.

[31] Al-Rashdi K.M., Claereboudt M.R., Al-Busaidi S.S. (2017). Density and Size Distribution of the Sea Cucumber, *Holothuria scabra* (Jaeger, 1935), at Six Exploited Sites in Mahout Bay, Sultanate of Oman. J. Agric. Mar. Sci. JAMS.

[32] Lavitra T., Rasolofonirina R., Grosjean P., Jangoux M., Eeckhaut I. (2010). The Effect of Food Quality and Rearing Density on the Growth and Survival of Epibenthic Juveniles of the Sea Cucumber *Holothuria scabra*. West. Indian Ocean. J. Mar. Sci..

[33] Eeckhaut I., Wayenberghe K.V., Nicolas F., Delroisse J. (2019). Skin Ulcerations in *Holothuria scabra* Can Be Induced by Various Types of Food. SPC Beche-De-Mer Inf. Bull..

[34] Delroisse J., Wayneberghe K.V., Flammang P., Gillan D., Gerbaux P., Opina N., Todinanahary G.G.B., Eeckhaut I. (2020). Epidemiology of a Skin Ulceration Disease (SKUD) in the Sea Cucumber *Holothuria scabra* with a Review on the SKUDs in Holothuroidea (Echinodermata). Sci. Rep..

[35] Bondoc K.G.V., Lee H., Cruz L.J., Lebrilla C.B., Juinio-Meñez M.A. (2013). Chemical Fingerprinting and Phylogenetic Mapping of Saponin Congeners from Three Tropical Holothurian Sea Cucumbers. Comp. Biochem. Physiol. Part B.

[36] Caulier G., Flammang P., Rakotorioa P., Gerbaux P., Demeyer M., Eeckhaut I. (2013). Preservation of the Bioactive Saponins of *Holothuria scabra* through the Processing of Trepang. Cah. Biol. Mar..

[37] Mercier A., Battaglene S.C., Hamel J.-F. (1999). Daily Burrowing Cycle and Feeding Activity of Juvenile Sea Cucumbers. J. Exp. Mar. Biol. Ecol..

[38] Plotieau T., Baele J.-M., Vaucher R., Hasler C.-A., Koudad D., Eeckhaut I. (2013). Analysis of the Impact of *Holothuria scabra* Intensive Farming on Sediment. Cah. Biol. Mar..

[39] Williamson J.E., Duce S., Joyce K.E., Raoult V. (2021). Putting Sea Cucumbers on the Map: Projected Holothurian Bioturbation Rates on a Coral Reef Scale. Coral Reefs.

[40] Wyatt T.D. (2013). Pheromones and Animal Behavior.

[41] Dal Bello M., Pérez-Escudero A., Schroeder F.C., Gore J. (2021). Inversion of Pheromone Preference Optimizes Foraging in *C. elegans*. eLife.

[42] Rono E., Njagi P.G.N., Bashir M.O., Hassanali A. (2008). Concentration-Dependent Parsimonious Releaser Roles of Gregarious Male Pheromone of the Desert Locust, *Schistocerca gregaria*. J. Insect Physiol..

[43] Hamel J.-F., Conand C., Pawson D.L., Mercier A. (2001). The Sea Cucumber *Holothuria scabra* (Holothuroidea: Echinodermata): Its Biology and Exploitation as Beche-de-Mer. Adv. Mar. Biol..

[44] Altamirano J.P., Recente C.P., Rodriguez J.C. (2016). Substrate Preference for Burying and Feeding of Sandfish *Holothuria scabra* Juveniles. Fish. Res..

[45] Schindelin J., Arganda-Carreras I., Frise E., Kaynig V., Longair M., Pietzsch T., Preibisch S., Schmid B., Tinevez J.-Y., White D.J. (2012). Fiji: An Open-Source Platform for Biological-Image Analysis. Nat. Methods.

[46] Rakotonjanahary F., Lavitra T., Fohy N., Eeckhaut I. (2016). Essais d’optimisation de La Croissance Des Juvéniles *d’Holothuria scabra* Pendant La Phase de Pré-Grossissement. Bêche-De-Mer Bull. D’Inf. CPS.

[47] Ahmed Q., Poot-Salazar A., Ali Q.M., Bat L. (2018). Seasonal Variation in the Length-Weight Relationships and Condition Factor of Four Commercially Important Sea Cucumbers Species from Karachi Coast-Northern Arabian Sea. Nat. Eng. Sci..

[48] Hamad M.I., Mwandya A.W., Munubi R.S., Chenyambuga S. (2019). Comparative Growth and Survival Performance of Sea Cucumber (*Holothuria scabra*) in Co-Cultured Pen System with Commercial Macroalgae. Afr. J. Biol. Sci..

[49] Juinio-Meñez M.A., Evangelio J.C., Miralao S.J.A. (2012). Trial Grow-out Culture of Sea Cucumber *Holothuria scabra* in Sea Cages and Pens. Aquac. Res..

[50] Juinio-Meñez M.A., Tech E.D., Ticao I.P., Gorospe J.R., Edullantes C.M.A., Rioja R.A.V. (2016). Adaptive and Integrated Culture Production Systems for the Tropical Sea Cucumber *Holothuria scabra*. Fish. Res..

[51] Baddeley A., Diggle P.J., Hardegen A., Lawrence T., Milne R.K., Nair G. (2014). On Tests of Spatial Pattern Based on Simulation Envelopes. Ecol. Monogr..

[52] Landler L., Ruxton G.D., Malkemper E.P. (2019). The Hermans–Rasson Test as a Powerful Alternative to the Rayleigh Test for Circular Statistics in Biology. BMC Ecol..

[53] Fitak R.R., Johnsen S. (2017). Bringing the Analysis of Animal Orientation Data Full Circle: Model-Based Approaches with Maximum Likelihood. J. Exp. Biol..

[54] Baddeley A., Turner R. (2005). Spatstat: An R Package for Analyzing Spatial Point Patterns. J. Stat. Softw..

[55] R Core Team (2020). R: A Language and Environment for Statistical Computing.

[56] Van den Spiegel D., Eeckhaut I., Jangoux M. (1998). Host Selection by *Synalpheus stimpsoni* (De Man), an Ectosymbiotic Shrimp of Comatulid Crinoids, Inferred by a Field Survey and Laboratory Experiments. J. Exp. Mar. Biol. Ecol..

[57] Eeckhaut I., McHugh D., Mardulyn P., Tiedemann R., Monteyne D., Jangoux M., Milinkovitch M.C. (2000). Myzostomida: A Link between Trochozoans and Flatworms?. Proc. R. Soc. Lond. Ser. B. Biol. Sci..

[58] Vaïtilingon D., Eeckhaut I., Fourgon D., Jangoux M. (2004). Population Dynamics, Infestation and Host Selection of Vexilla Vexillum, an Ectoparasitic Muricid of Echinoids, in Madagascar. Dis. Aquat. Org..

[59] Fourgon D., Jangoux M., Eeckhaut I. (2007). Biology of a “Babysitting” Symbiosis in Brittle Stars: Analysis of the Interactions between Ophiomastix venosa and *Ophiocoma scolopendrina*. Invertebr. Biol..

[60] Caulier G., Mezali K., Soualili D.L., Decroo C., Demeyer M., Eeckhaut I., Gerbaux P., Flammang P. (2016). Chemical Characterization of Saponins Contained in the Body Wall and the Cuvierian Tubules of the Sea Cucumber *Holothuria (platyperona) sanctori* (Delle Chiaje, 1823). Biochem. Syst. Ecol..

[61] Van Dyck S., Gerbaux P., Flammang P. (2009). Elucidation of Molecular Diversity and Body Distribution of Saponins in the Sea Cucumber *Holothuria forskali* (Echinodermata) by Mass Spectrometry. Comp. Biochem. Physiol. Part B.

[62] Decroo C., Colson E., Demeyer M., Lemaur V., Caulier G., Eeckhaut I., Cornil J., Flammang P., Gerbaux P. (2017). Tackling Saponin Diversity in Marine Animals by Mass Spectrometry: Data Acquisition and Integration. Anal. Bioanal. Chem..

