# Supplementary material for: A Distinct Saponin Profile Drives an Olfactory-Mediated Aggregation in the Aquacultivated Sea Cucumber Holothuria scabra
Source: Mar Drugs. 2023 Mar 16;21(3):184. doi: 10.3390/md21030184 (PMC10053547; doi:10.3390/md21030184)
Supplement: Supplementary file 1 [file marinedrugs-21-00184-s001.zip › marinedrugs-2254321-supplementary.pdf]

marine drugs

**Supplementary Figure S1**

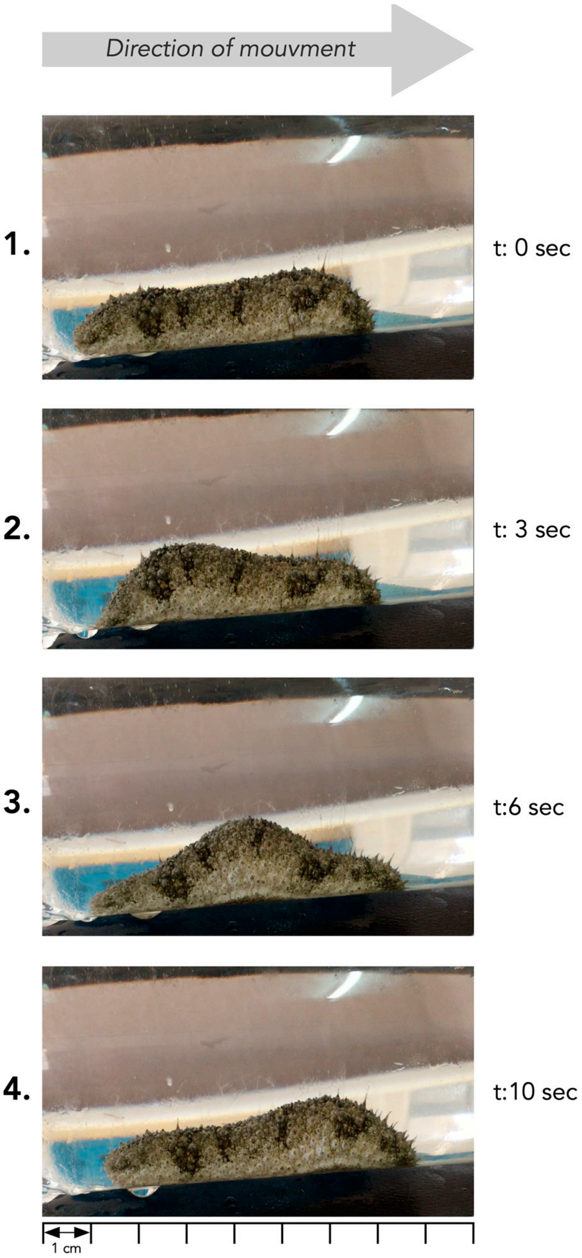


**Figure S1.** Locomotion of a stimulated *H. scabra* juvenile in a glass Y-tube. The locomotion began with the detachment of the terminal quarter of the body's length (1), the body then contracted and thickened in diameter (2) while the front 3/4 of the body remained thin. A peristaltic wave then progressed from the posterior part to the anterior part, increasing the diameter of the body at the wave progressed (3, 4). At the level of this peristaltic wave, the podia of the trivium detach themselves from the substrate to reattach themselves to it after the wave. The overall effect of this locomotion was that the animal progressed by about a quarter of its body-length after each peristaltic wave.
